# Supplementary material for: The Large GTPase, GBP-2, Regulates Rho Family GTPases to Inhibit Migration and Invadosome Formation in Breast Cancer Cells
Source: Cancers (Basel). 2021 Nov 11;13(22):5632. doi: 10.3390/cancers13225632 (PMC8616281; doi:10.3390/cancers13225632)

cancers

Supplementary Materials

The large GTPase, GBP-2, Regulates Rho Family GTPases to Inhibit Migration and Invadosome Formation in Breast Cancer Cells

Geoffrey O. Nyabuto, John P. Wilson, Samantha A. Heilman, Ryan C. Kalb, Jonathan P. Kopacz, Ankita V. Abnave and Deborah J. Vestal

Method for Supplemental Figure S1: Cells were plated in duplicates in 6-well dishes and incubated for 24 hrs at 37°C to confluence. A 200 µL pipette tip was used to create a scratch and the debris was removed with a PBS wash. Three fields per well were photographed immediately (0 hr) and 24 hr after scratch formation on an EVOS FL Inverted Microscope (Thermofisher) at 4× [43]. The wound width at 0 hr and 24 hrs post-introduction of scratch was measured using MetaMorph (Molecular Devices, San Jose, CA, USA). The difference between wound width at 24 hrs and at 0 hr was used to calculate the percent of the wound closure in relation to wound width at 0 hr.

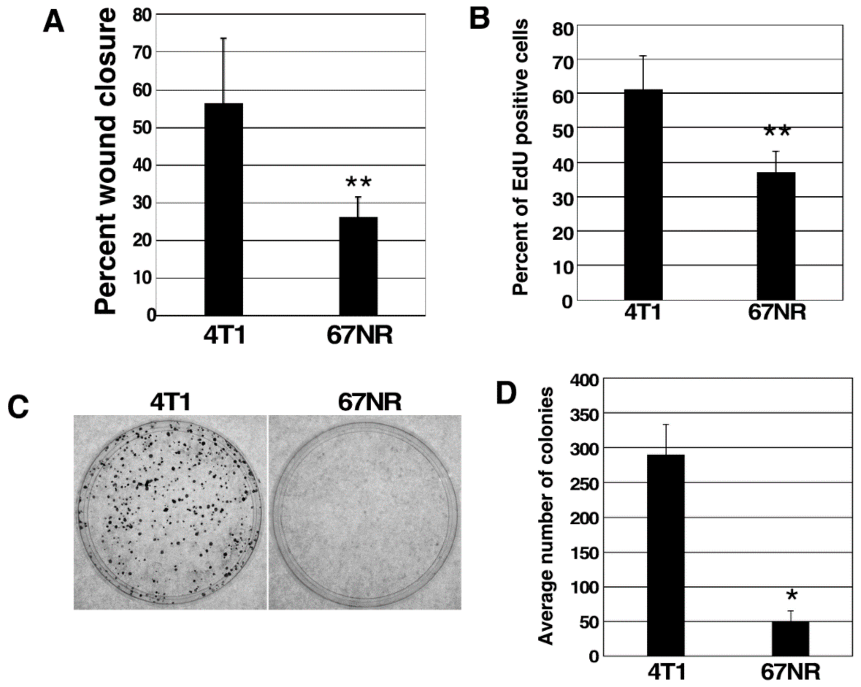

Figure S1. 4T1 cells migrate significantly faster than 67NR cells; (**A**) Confluent 4T1 and 67NR cells were scratch with a 200 µL pipette tip and wound closure was analyzed as described in Supplemental Figure Methods. Wound closure was presented as average closure ± standard deviation (SD) (** = p <0.01 compared to 4T1 cells, n = 3). **(C)** 4T1 (3×10^5 cells/coverslip) and 67NR cells (4×10^5 cells/coverslip) were cultured in duplicates in 6-well dishes and analyzed for S phase cells as described. The graph depicts the average percentage of EdU positive cells + SD (** = p <0.01 compared to 4T1 cells, n = 3). **(D)** 4T1 and 67NR cells (1×10^3 cells/dish) were seeded in triplicate in 6-cm dishes. After 6 days, cells were fixed and stained with crystal violet. Representative images are shown. (**E**). All of the colonies with greater than 50 cells were counted per plate and represented as the average number of colonies per cell line + SD (* = p <0.05 compared to 4T1 cells, n = 3).

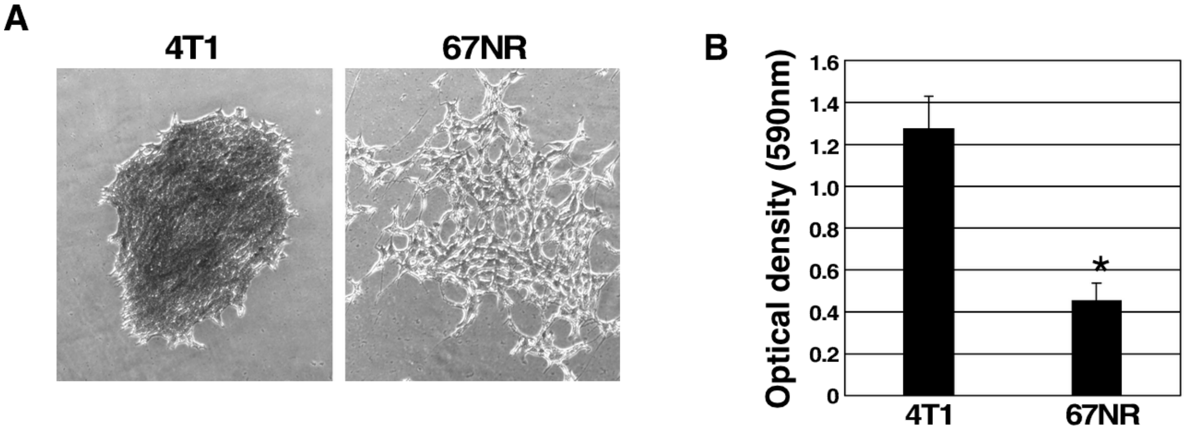

Figure S2. 4T1 cells have more proliferating cells than 67NR cells. (**A**) Representative photomicrographs (40×) of a single colony of 4T1 and 67NR cells show differences in colony morphology. (**B**). Crystal violet was extracted from the plates in part D with 1% SDS and quantified at 590 nm. The average optical densities ± SD are shown (* = p <0.05 compared to 4T1 cells, n = 3).

The uncropped autorads for the Western blots follow:

2A

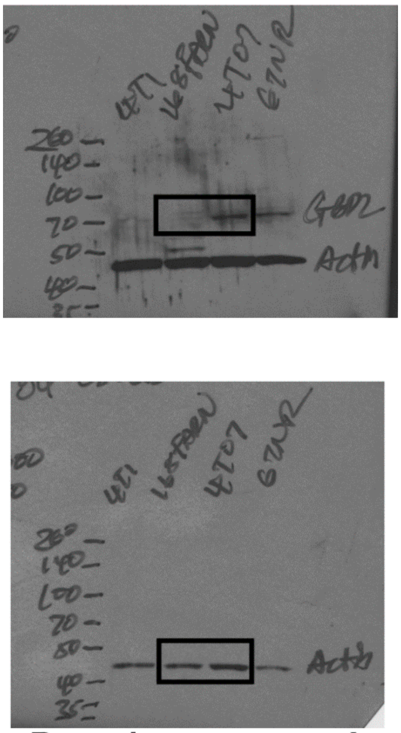

Box = lanes removed

2B

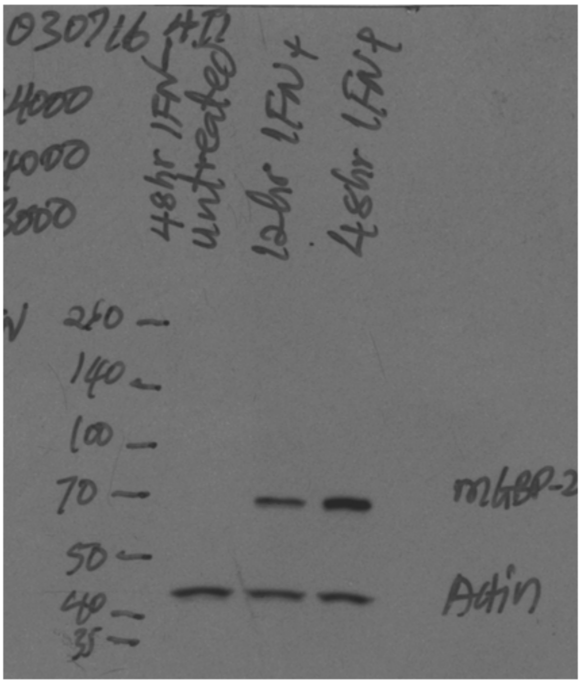

2F

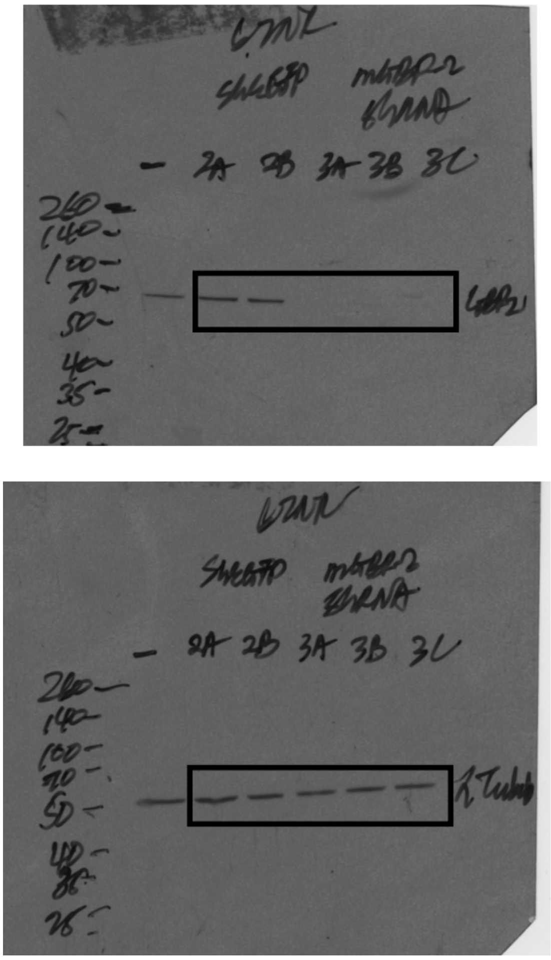

3C

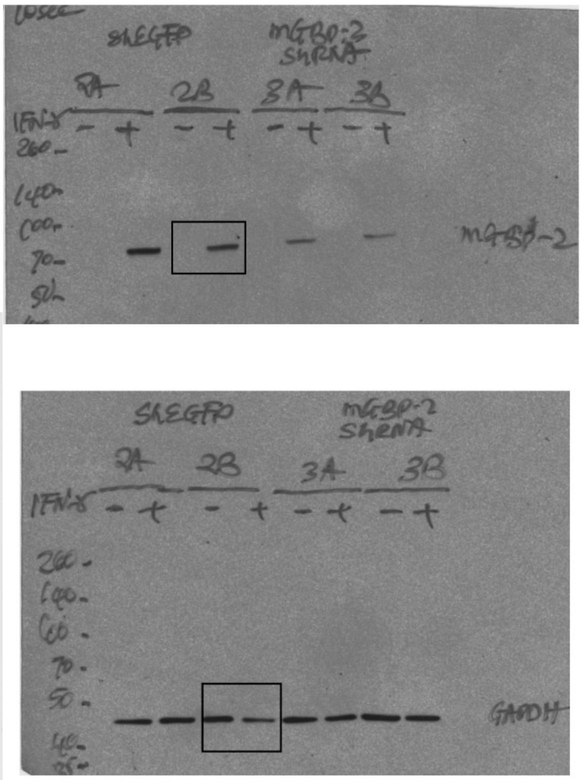

Box = area used

The area within the black box was cropped out of the figure.

4A

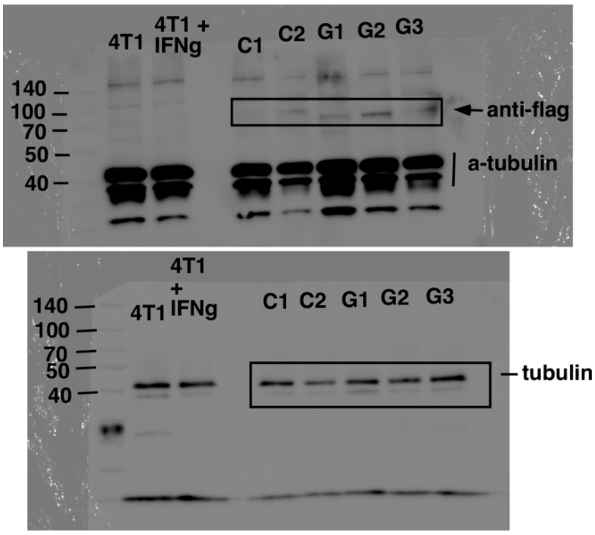

4B

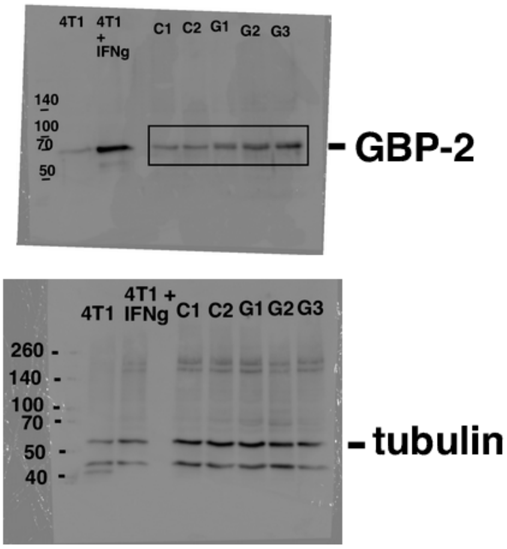

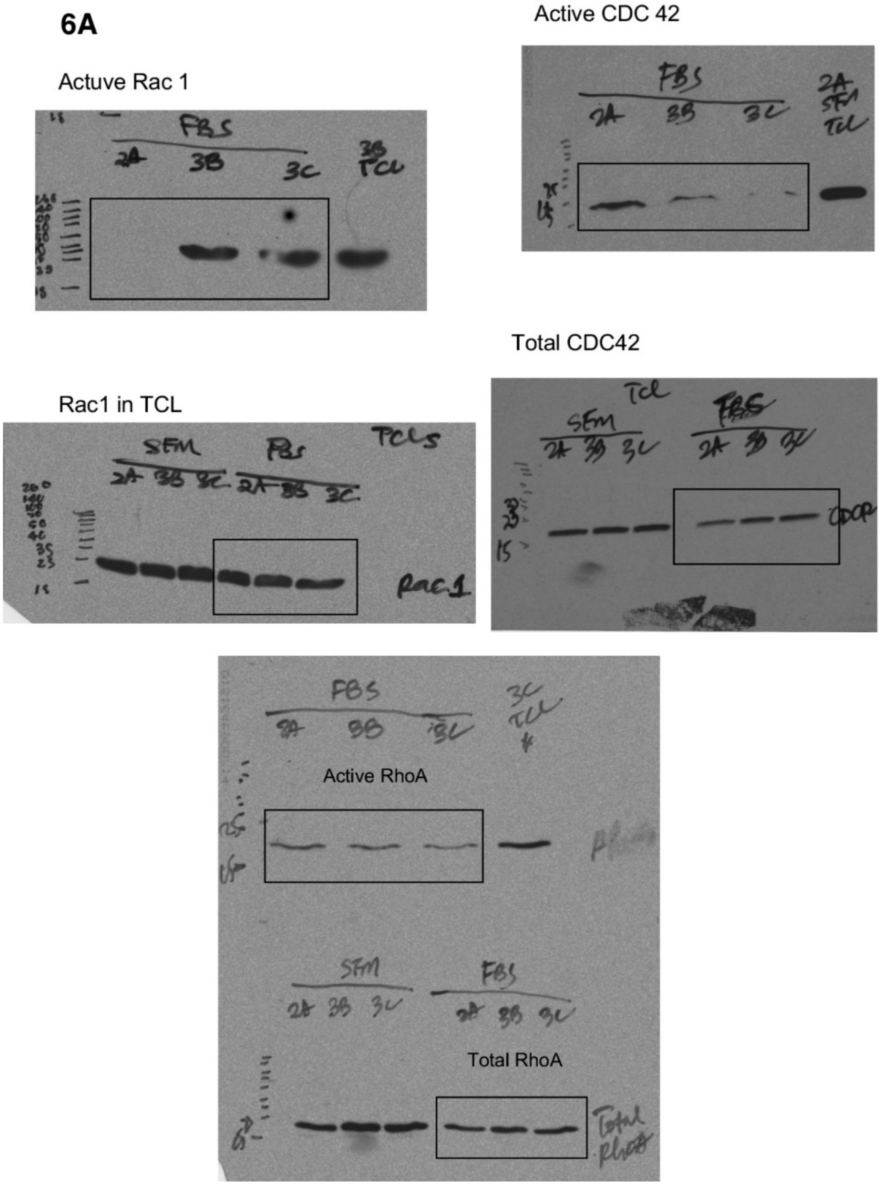
6A
Actuve Rac 1
Active CDC 42
Total CDC42
Rac1 in TCL
Active RhoA
Total RhoA

6E

phospho-Akt

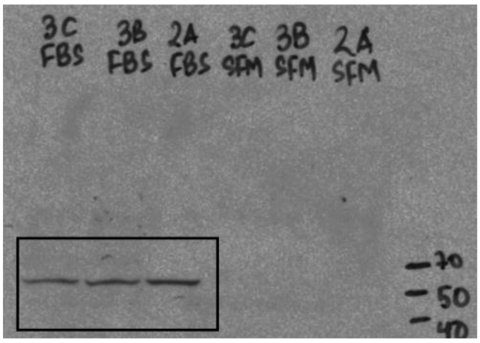

Total Akt

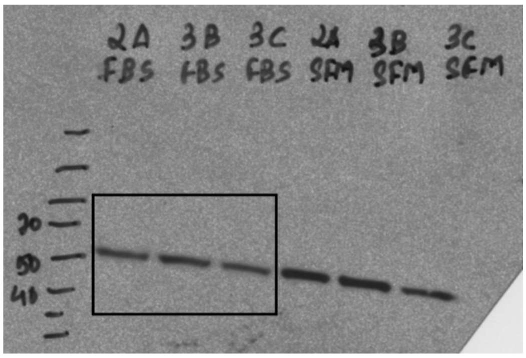

Supplement: Supplementary file 1 [file cancers-13-05632-s001.zip › cancers-1464984-supplementary.pdf]
